# Epidermal Growth Factor-Like Domain-Containing Protein 7 (EGFL7) Enhances EGF Receptor−AKT Signaling, Epithelial−Mesenchymal Transition, and Metastasis of Gastric Cancer Cells

**DOI:** 10.1371/journal.pone.0099922

**Published:** 2014-06-19

**Authors:** Bai-Hua Luo, Feng Xiong, Jun-Pu Wang, Jing-He Li, Ming Zhong, Qin-Lai Liu, Geng-Qiu Luo, Xiao-Jing Yang, Ni Xiao, Bin Xie, Heng Xiao, Rui-Jie Liu, Chang-Sheng Dong, Kuan-Song Wang, Ji-Fang Wen

**Affiliations:** 1 Department of Pathology, Xiangya Hospital, Central South University, Changsha, Hunan, China; 2 Department of Pathology, School of Basic Medical Sciences, Central South University, Changsha, Hunan, China; 3 Center of Furong Judicial Authentication, the Second People’s Hospital, Hunan, China; 4 Department of Pathology, Taishan Medical College, Taian, Shandong, China; 5 Department of anesthesiology, Affiliated Tumor Hospital of Xiangya Medical College, Central South University, Changsha, Hunan, China; Vanderbilt University, United States of America

## Abstract

Epidermal growth factor-like domain-containing protein 7 (EGFL7) is upregulated in human epithelial tumors and so is a potential biomarker for malignancy. Indeed, previous studies have shown that high EGFL7 expression promotes infiltration and metastasis of gastric carcinoma. The epithelial–mesenchymal transition (EMT) initiates the metastatic cascade and endows cancer cells with invasive and migratory capacity; however, it is not known if EGFL7 promotes metastasis by triggering EMT. We found that EGFL7 was overexpressed in multiple human gastric cancer (GC) cell lines and that overexpression promoted cell invasion and migration as revealed by scratch wound and transwell migration assays. Conversely, shRNA-mediated EGFL7 knockdown reduced invasion and migration. Furthermore, EGFL7-overexpressing cells grew into larger tumors and were more likely to metastasize to the liver compared to underexpressing CG cells following subcutaneous injection in mice. EGFL7 overexpression protected GC cell lines against anoikis, providing a plausible mechanism for this enhanced metastatic capacity. In excised human gastric tumors, expression of EGFL7 was positively correlated with expression levels of the mesenchymal marker vimentin and the EMT-associated transcription repressor Snail, and negatively correlated with expression of the epithelial cell marker E-cadherin. In GC cell lines, EGFL7 knockdown reversed morphological signs of EMT and decreased both vimentin and Snail expression. In addition, EGFL7 overexpression promoted EGF receptor (EGFR) and protein kinase B (AKT) phospho-activation, effects markedly suppressed by the EGFR tyrosine kinase inhibitor AG1478. Moreover, AG1478 also reduced the elevated invasive and migratory capacity of GC cell lines overexpressing EGFL7. Collectively, these results strongly suggest that EGFL7 promotes metastasis by activating EMT through an EGFR−AKT−Snail signaling pathway. Disruption of EGFL7−EGFR−AKT−Snail signaling may a promising therapeutic strategy for gastric cancer.

## Introduction

Gastric cancer (GC) is the fourth most common malignant tumor and the second leading cause of cancer-related mortality worldwide [1], [2]. Approximately half of all GC cases occur in East Asian countries, with particularly high incidences in Japan, Korea, and China [3], [4]. Progress in the systemic treatment of GC has greatly increased short-term survival; however, the five-year survival rate of GC patients remains low due to relapse and metastasis [5]. Moreover, most newly diagnosed GC patients already show metastatic disease, which constitutes a major therapeutic challenge for oncologists [6].

Epidermal growth factor-like domain-containing protein 7 (EGFL7), also known as vascular endothelial statin, is an endothelial cell-derived secreted factor that regulates vascular tube formation. Parker et al. [7] demonstrated that EGFL7 is crucial for angiogenesis during zebra fish embryogenesis [8]. Recent studies have reported elevated expression of EGFL7 in several tumors and cancer cell lines, including kidney tumors, malignant gliomas, hepatocellular carcinomas, and colon cancers [7]−[10]. We previously demonstrated that EGFL7 is also overexpressed in gastric carcinoma [11], and expression was significantly correlated with pathologic characteristics, clinical progression, poor prognosis, and metastasis [10], [12]. Therefore, EGFL7 is a candidate predictive factor for cancer progression and metastasis. However, the mechanisms underlying the tumorigenic effects of EGFL7 are unclear.

Metastasis is a multi-step process that involves an epithelial–mesenchymal transition (EMT) in which polarized epithelial cells are converted to mesenchymal cells [13], a phenotype with greater invasive and migratory capacity [14]. The EMT is also a reversible process that often occurs at the invasive front of many metastatic cancers [15]. Several studies have shown that EGF promotes cancer cell migration and invasion concomitant with activation of EMT [16]−[18]. Significantly, EGFL7 contains two EGF-like domains, suggesting some functional homology [9], [12]. However, whether EGFL7 actually does enhance EMT and promote gastric cancer metastasis has yet to be determined. Moreover, the molecular mechanisms by which EMT is regulated in GC remain largely unknown. The zinc finger transcriptional repressor Snail is critical for gene expression reprogramming during EMT, notably for repression of the cell−cell adhesion protein endothelial (E)-cadherin, the loss of which is considered an important early event in EMT and necessary for subsequent metastasis [19].

The present study aimed to determine if EGFL7 promotes metastasis by triggering EMT. We found that EGFL7 overexpression activates the EGFR−AKT pathway, triggers EMT, and promotes GC cell invasion *in vitro* and metastasis *in vivo*.

## Materials and Methods

### Patients and Tissue Collection

Gastric carcinoma tissues were obtained from 79 GC patients (58 males and 21 females) treated by surgical resection at the Department of Surgery, Xiangya Hospital, Central South University (China). Surgeries were conducted from Jan 2010 to Dec 2012. The patients were 54.7 years old on average (range: 28 to 82 years) and received neither chemotherapy nor radiation therapy before surgery.

The patients were informed that surgical specimens would be used for conventional pathological diagnosis and that the remaining tissues could be used for research. No personal patient data was required for this study and the protocols carried no risk, so only verbal informed consent was required from the patients. The protocol was approved by the Ethics Committee of Xiangya Hospital, Central South University (Permit Number: 201311389).

Disease staging was defined according to the sixth edition of the TNM staging system of the American Joint Committee on Cancer [20], [21]. The tumors were well differentiated gastric adenocarcinoma in 12 cases, moderately differentiated in 34 cases, and poorly differentiated in 33 cases. All samples were immediately fixed with 10% formalin, dehydrated, and paraffin-embedded. Accurate clinical information and pathologic diagnosis were available for all patients.

### Immunohistochemistry

For immunohistochemistry, prepared samples were incubated at 60°C for 15 h and cut into 4 µm–thick sections, deparaffinized in turpentine, dried, and rehydrated in a decreasing ethanol: distilled water gradient. Endogenous peroxidase activity was inactivated by incubation for 30 min in 0.3% hydrogen peroxide in 0.01 M phosphate-buffered saline (PBS). Slices were then incubated for 15−20 min in citrate buffer (pH 6.0) at 95°C for antigen retrieval, blocked with 5% normal goat serum in 0.01 M PBS for 15 min at 37°C, and incubated with primary antibodies diluted in blocking solution overnight at 4°C in a humidified chamber. The following primary antibodies were used: mouse anti-EGFL7 monoclonal antibody (Abcam, USA, 1∶400) and rabbit monoclonal antibodies raised against E-cadherin (CST, USA, 1∶1000), vimentin (CST, USA, 1∶1000), Snail (Proteintech, USA, 1∶1000), and CD34 (Boster, China, 1∶500). After washing, samples were successively incubated with biotinylated secondary antibody and streptavidin–horseradish peroxidase (HRP) avidin working solution. Immunolabeling was visualized using the DAB substrate kit from Zhongshan Golden Bridge Company (China) according to the manufacturer’s recommendations. Finally, the sections were counterstained with hematoxylin (Vector Laboratories, USA), dehydrated in an ascending ethanol:distilled water gradient, and mounted on slides using Permount mounting media (Fisher Scientific, USA).

### Quantification of Immunohistochemical Signals

All GC and surrounding non-neoplastic gastric tissues were confirmed histopathologically by two independent pathologists (K.-S.W. and J.-H.L.) blind to the original diagnosis. Immunostaining was graded by a semi-quantitative method that considered both the intensity and distribution of the staining [22]. Briefly, five fields were randomly selected under low magnification (100×, Olympus BX51, Tokyo, Japan), and 200 tumor cells counted from each field. The staining intensity was scored as follows: 0, no staining; 1, weak yellow staining; 2, yellow staining; 3, brown staining. Positive cells were characterized by a clear border and yellow or brown staining distinct from the background. The percentage of positive cells (PP) was scored as follows: 0, 0%; 1, 1%−25%; 2, 26%−50%; 3, 51%−75%; 4, 76%−100%. The two scores were combined to obtain the following classification: negative staining, samples with immunoreactive score (IRS) of 0 to 2; weakly positive staining, samples with IRS of 3 to 5; strongly positive staining, samples with IRS of 6 to 7. Samples that showed weakly and strongly positive staining were included in the statistical analysis.

### Cell Lines and Culture

Human GC cell lines SGC7901 (moderately differentiated adenocarcinoma), BGC823 (poorly differentiated adenocarcinoma), MKN28 (well-differentiated adenocarcinoma), and MKN45 (poorly differentiated adenocarcinoma) as well as normal gastric mucosa epithelial GES-1 cells were purchased from the Type Culture Collection of the Chinese Academy of Sciences (Shanghai, China). All cells were cultured and maintained in Roswell Park Memorial Institute (RPMI) 1640 medium (Gibco Biocult, Paisley, UK) supplemented with 10% fetal bovine serum (FBS) (Gibco Biotechnology), 100 U/ml penicillin, and 100 µg/ml streptomycin at 37°C in a humidified atmosphere containing 5% CO_2_.

### Short Hairpin RNA (shRNA) and Recombinant Expression Plasmid Pex-2-EGFL7

To generate cell lines overexpressing or underexpressing EGFL7, the native cell lines were stably transfected with an expression vector or targeted shRNA. The shRNA sequences of human EGFL7 were designed according to the human EGFL7 DNA sequence (GenBank NO. NM_016215.3) by Designer 3.0 software (Genepharma, http://www.genepharma.com) and BLAST searches (http://www.ncbi.nlm.nih.gov/BLAST). The target sequences were compared to the human genome database by BLAST search to ensure no high homology to other coding sequences. The sequences EGFL7-homo-333 and EGFL7-homo-887 were identified as target sites for a specific EGFL7 shRNA. In addition, one nonspecific sequence (scramble shRNA) was designed as a negative control, and the GAPDH sequence was designed as an internal control. The shRNA sequences were as follows:

EGFL7-shRNA1 against EGFL7-homo-333: sense, 5′-CACCGGTGCTGCTGATGTGGCTTTCAAGAGAAGCCACATCAGCAGCACCTTTTTTG-3′; antisense, 5′-GATCCAAAAAAGGTGCTGCTGATGTGGCTTCTCTTGAAAGCCACATCAGCAGCACC-3′; EGFL7-shRNA2 against EGFL7-homo-887: sense, 5′-CACCGAGTGGACAGTGCAATGAATTCAAGAGATTCATTGCACTGTCCACTCTTTTTTG-3′; antisense, 5′-GATCCAAAAAAGAGTGGACAGTGCAATGAATCTCTTGAATTCATTGCACTGTCCACTC-3′; nonspecific sense, 5′-CACC GTTCTCCGAACGTGTCACGTCAAGAGATTACGTGACACGTTCGGAGAATTTTTTG-3′; antisense, 5′-GATCCAAAAAATTCTCCGAACGTGTCACGTAATCTCTTGACGTGACACGTTCGGAGAAC-3′; GAPDH sense, 5′-CACCGTATGACAACAGCCTCAAGTTCAAGAGA CTTGAGGCTGTTGTCATACTTTTTTG-3′; antisense, 5′-GATCCAAAAAAGTATGACAACAGCCTCAAGTCTCTTGAACTTGAGGCTGTTGTCATAC-3′. The pGPU6/GFP/Neo (green fluorescent protein, neomycin) vector (Genepharma Company, Shanghai, China) was used for plasmid construction. The nucleotides were annealed and inserted into the BamHI and HindIII sites and transformed into DH5a-competent E. coli. Dual kanamycin/neomycin-resistant colonies were selected and the vector sequences confirmed by restriction digestion and DNA sequencing.

The EGFL7 cDNA encoding the 533−1353 amino acid sequence was subcloned into a pEX-2 plasmid vector (Genepharma, Shanghai, China) by BglII/EcoRI double digestion and designated pEX-2-EGFL7. The recombinant plasmid was amplified in DH5a-competent E. coli and the vector sequence amplified by PCR. DNA sequencing confirmed that the recombinant plasmid contained an EGFL7 gene fragment identical to that in GenBank (GenBank NO. NM_016215.3). Kanamycin/neomycin-resistant colonies were selected and the sequence confirmed by restriction digestion and DNA sequencing. All primers were designed and synthesized by Genepharma (Shanghai, China).

### Stable Transfection and G418 Selection

To establish an EGFL7-underexpressing clone (and appropriate control), BGC823 cells were seeded in six-well culture plates at 1×10^6^/well and incubated for 24 h in RPMI 1640 medium containing 10% FBS in a humidified 5% CO_2_ atmosphere maintained at 37°C. The cells were transfected with 4 µg pGPU6/GFP/Neo-EGFL7-shRNA1, pGPU6/GFP/Neo-EGFL7-shRNA2, pGPU6/GFP/Neo-nonspecific-shRNA, or pGPU6/GFP/Neo-GAPDH-shRNA using Lipofectamine 2000 transfection reagent (Invitrogen, Carlsbad, CA, USA) following the manufacturer’s instructions. After 24 h, cells were incubated in G418 (Sigma, USA, 600 µg/ml) for initial selection, diluted, plated at low density (∼1 cell/well) and maintained in culture medium supplemented with G418 at half the initial selection concentration for an additional 3 weeks. The resulting stable cell lines were named BGC2-13 (cell line resulting from pGPU6/GFP/Neo-EGFL7-shRNA1 stable transfection) and BGC-NC, and expanded for subsequent studies. The expression level of EGFL7 was evaluated by Western blot and real-time RT-PCR. To establish an overexpressing line and matched control, MKN28 cells were seeded, transfected with 4 µg of pEX-2-EGFL7 or pEX-2 plasmids, and selected as described for BGC823 cells except that 500 µg/ml G418 was used for initial selection. The resulting stable cell lines were named MKN28-EGFL7 and MKN28-NC. The expression levels of EGFL7 in G418-resistant clones were evaluated by Western blot and quantitative real-time PCR (qRT-PCR).

### Western Blot Analysis

After cells reached 80%−95% confluence, total protein was extracted using cell lysate extraction buffer (Beyotime, China) containing protease inhibitor (1 mM phenylmethylsulfonyl fluoride). Lysate total protein concentration was determined using a bicinchoninic acid protein assay kit (Pierce Biotechnology, Rockford, IL, USA). Equal amounts of protein (25 µg) from each treatment group were separated per gel lane by 8% sodium dodecyl sulfate–polyacrylamide gel electrophoresis (SDS-PAGE) and transferred to polyvinylidene difluoride (PVDF) membranes (Millipore, Billerica, USA). The membranes were sequentially incubated with blocking buffer consisting of Tris-buffered saline (TBS) containing 5% nonfat dry milk for 2 h at room temperature and then overnight at 4°C in this same buffer with one of the following primary antibodies: anti-EGFL7 (1∶500; Abcam Cambridge Science, UK). anti-E-cadherin, anti-vimentin, anti-Snail (all at 1∶1000; Cell Signaling Technology, CST, USA), anti-GAPDH (1∶10000, Protech), anti-EGFR, anti-phospho-EGFR, anti-AKT, anti-phospho-AKT, anti-ERK, and anti-phospho-ERK (all 1∶800, Anbo Biotech Co., Ltd., USA). Immunolabeled membranes were then incubated with a HRP-conjugated secondary antibody (1∶2000, Immunology Consultants Laboratory, Inc., USA) for 2 h at room temperature. After washing with TBST, protein bands were visualized using the enhanced chemiluminescence detection system (Advansta Corporation, Menlo Park, California, USA) according to the manufacturer’s instructions. Expression of GAPDH was used as a loading control and proteins quantified using UTHSCSA Image Tool 3.0. Target protein expression was calculated as the band intensity ratio of the target protein to GAPDH.

### RNA Extraction and PCR Analysis

Cells were lysed in TRIzol reagent (Invitrogen, Carlsbad, CA, USA) and total RNA was prepared according to the manufacturer’s instructions. First strain cDNAs were synthesized by the PrimeScript RT reagent Kit with gDNA Eraser (Takara, Otsu, Japan) and amplified by PCR using the following specific primers: EGFL7 sense, 5′-GACCCTGTCTCCGAGTCGTTC-3′; antisense, 5′-GATGGTTCGGTAGGTGCTGC-3′. The cDNA of GAPDH was amplified to control for the amount of cDNA present in each sample (sense, 5′-GCACCGTCAAGGCTGAGAAC-3′; antisense, 5′-TGGTGAAGACGCCAGTGGA-3′). PCR amplification was conducted at 94°C for 5 min, followed by 30 cycles of 94°C for 30 s, 57°C for 30 s, and 72°C for 40 s. PCR products were separated on 1.0% agarose gels and target gene expression quantified as the ratio of the target gene band intensity to that of GAPDH using Image Tool 3.0 (The University of Texas Health Science Center, San Antonio, Texas, USA).

### Real-time PCR

The reaction mixture for real-time PCR consisted of 10 µl of Premix Ex Taq (Probe qPCR), 0.2 µM of each EGFL7 and GAPDH primer (below), 0.2 pmol/ml TaqMan probes (EGFL7 or GAPDH), and 0.4 µl ROX (tetrapropano-6-carboxyrhodamine) Reference Dye II (Takara Bio, Shiga, Japan). The mixture was combined with 2 µl cDNA in each well of a 96-well MicroAmp plate (Applied Biosystems, CA, USA) and distilled water was used to adjust to a final volume of 20 µl. The following primers were designed using the Primer Premier 6.0 software package (Premier Biosoft International, Palo Alto, CA, USA): EGFL7: forward, 5′-GACCCTGTCTCCGAGTCGTTC-3′; reverse, 5′-GATGGTTCGGTAGGTGCTGC-3′; GAPDH: forward, 5′-GCACCGTCAAGGCTGAGAAC-3′; reverse, 5′-TGGTGAAGACGCCAGTGGA-3′. All reactions were performed on a 7500 Real-Time PCR System (Applied Biosystems, CA, USA). Thermocycle conditions were initial denaturation at 95.8°C for 30 s followed by 40 cycles of amplification at 95.8°C for 6 s and 60.8°C for 30 s.

Similar experimental conditions were used to assess the expression of other genes with the following primers: E-cadherin: forward, 5′-GAGTGCCAACTGGACCATTCAGTA-3′; reverse, 5′-AGTCACCCACCTCTAAGGCCATC-3′; vimentin: forward, 5′-GACGCCATCAACACCGAGTT-3′; reverse, 5′-CTTTGTCGTTGGTTAGCTGGT-3′; Snail: forward, 5′-TGCTCCACAAGCACCAAGA-3′; reverse, 5′-GCAGAGGACACAGAACCAGAAA-3′; GAPDH: forward, 5′-GCACCGTCAAGGCTGAGAAC-3′; reverse, 5′-TGGTGAAGACGCCAGTGGA-3′. The reaction mixture for real-time PCR consisted of 10 µl of SYBR Premix Ex TaqTM II, 1.6 µl of each primer (0.4 µM), 0.4 µl of ROX Reference Dye, 2 µl of template cDNA, and 6 µl of diethyl pyrocarbonate-treated water. Real-time PCR was performed on a 7500 Real-Time PCR System (Applied Biosystems, CA, USA) with the following thermocycle conditions: initial denaturation at 95°C for 10 s, followed by 40 cycles of amplification at 95°C for 5 s and 60°C for 34 s.

### Cell Proliferation Assay

Cell proliferation was estimated by the 3-[4,5-dimethylthiazol-2-yl]-2,5 diphenyl tetrazolium bromide (MTT) viable cell assay. Cells were plated on 96-well plates at 2000/well and cultured for 12, 24, 48, 72, and 96 h. Then, 20 µl of MTT (Sigma) stock solution (5 mg/ml) was added to 200 µl of medium in each well, and plates were incubated for an additional 4 h at 37°C. After careful aspiration of the culture medium, 150 µl dimethyl sulfoxide (DMSO) was added to each well to dissolve formazan crystals formed from MTT by viable cells. The plates were shaken on a rotary platform for 10 min and absorbance measured at 490 nm using a microplate reader.

### Plate Colony Formation Assay

Cells were seeded at 200/well in the same six-well plates as used for other assays to assess colony formation under cell-adherent conditions. After 10 days, cells were stained with 1% Giemsa, and the number of visible colonies was counted. The relative clone formation index was calculated as mean number of colonies/number of seeded cells×100%.

### Scratch Wound Healing Assays

To assay cell migration, 5×10^5^ cells/well were plated in six-well plates coated with 10 µg/ml fibronectin (FN) and incubated for 24 h. The monolayer was then disrupted by a single scratch using a 200 µl pipette tip. Micrographs were obtained at 0, 12, 24, 36, 48, and 72 h post-scratch with a phase-contrast microscope (Olympus BX51, Japan). The number of cells within the scratched (barren) region of the microwell was counted in five fields (Magnification: ×200). All experiments were performed in duplicate.

### *In vitro* Invasion and Migration Assay

Cell invasion and migration were evaluated using transwell chambers (Corning, New York, USA). Invasion assays were conducted in transwell chambers separated by polycarbonate membrane filter inserts (8 µm pores) for 24-well plates. Each chamber was coated with 100 µl of 1∶20 Matrigel (Becton, Dickinson and Company, New York, USA) in cold RPMI 1640 overnight at 4°C. Subsequently, cells (5×10^4^/ml×200 µl) were seeded in the upper chamber in serum-free medium. Approximately 800 µl of medium conditioned with 10 µg/ml fibronectin was placed in the lower compartment of the transwell chamber as a chemoattractant. After incubation for 24 h at 37°C, the remaining tumor cells on the upper surface of the chamber were removed by wiping with wet cotton swabs. Invading cells on the lower surface were fixed with 4% paraformaldehyde, stained with crystal violet, and counted under a phase-contrast microscope (Olympus, Japan) at ×200. Four independent experiments were performed in triplicate for all treatment conditions. *In vitro* migration assays were conducted under the same conditions as the invasion assays, but with uncoated lower chambers and using 1×10^5^ cells/ml (200 µl).

### Anoikis Assay

Poly-hydroxyethyl methacrylate (poly-HEMA, Sigma-Aldrich), which inhibits cell adhesion to culture plates and other growth surfaces, was reconstituted in 95% ethanol to a final concentration of 12 mg/ml. To prepare poly-HEMA–coated plates, 2 ml of this solution was added to each well of a 6-well plate and allowed to dry overnight under a laminar flow tissue culture hood. Then 5×10^4^ cells were plated in triplicate in each poly-HEMA–coated well with regular culture media. After 24 h, GC cells were harvested, rinsed with PBS, and resuspended in 500 µl binding buffer from an Annexin V-PE/7-AAD Apoptosis Detection Kit (eBioscience, San Diego, USA). Resuspended cells from each sample were aliquoted (100 µl) into four tubes. Three tubes were labeled with either Annexin V-PE, 7-AAD, or both, while the fourth was used as an unlabeled control. Each batch was then analyzed by flow cytometry on a Beckman Coulter FC 500 counter (Beckman Coulter, Inc.). The percentage of apoptotic cells was derived as the sum of cell fractions displaying early apoptosis (annexin V-positive) and late apoptosis (7-AAD-positive).

### Xenograft Nude Mouse Model

Xenografting of GC cell lines was performed according to the National Guidelines for the Care and Use of Laboratory Animals (publication no. 86-23, revised 1985), was approved by the Animal Care and Use Committee of the Third Xiangya Hospital, Central South University (LLSC [LA] 2013-0010), and conformed to current Chinese law on the protection of animals (LLSC [LA] 2013-0010). All efforts were made to minimize the number of mice used and their suffering.

Thirty-five female Balb/c nude mice age 4 to 6 weeks were purchased from Slac Laboratory Animal Co. Ltd. (Shanghai, China) and housed in individually ventilated cages. Mice were randomly divided into BGC823, BGC-NC, BGC2-13, MKN28, MKN28-NC, MKN28-EGFL7, and control PBS groups (n = 5 mice/group). Cultured cells were harvested and resuspended in PBS at 1×10^8^ cells/0.2 ml. The suspension was injected subcutaneously into the left upper extremity of each nude mouse except for those in the control group, which were injected with 0.2 ml PBS. Tumor growth was evaluated every 3 days by measuring tumor diameters with Vernier calipers. Tumor volume (TV) was calculated according to the formula: TV (mm^3^) = d^2^×D/2, where d and D are the shortest and the longest diameters, respectively. The mice were sacrificed at 4 weeks after cell implantation, and the tumors were extracted and weighed. All livers were examined for metastasis by hematoxylin and eosin (H&E) staining. Tumors were fixed in 10% formalin, embedded in paraffin, and cut into 4 µm–thick sections. EGFL7 expression was determined by immunohistochemistry as described above.

### Statistical Analyses

All statistical analyses were performed using SPSS Version 16.0 (SPSS Inc., Chicago, IL, USA) for Windows. Data are expressed as mean ± standard deviation (SD) from at least three independent experiments. Group means were compared by one-way analysis of variance (ANOVA) with Student-Newman-Keuls (SNK) or least significant difference (LSD) tests for post hoc pair-wise comparisons. The Spearman Rank Correlation Coefficient was used to determine the correlations between EGFL7 and EMT-related protein expression levels in surgically excised gastric cancer tissues. A *P*<0.05 was considered statistically significant.

## Results

### EGFL7 Expression was Elevated in Human Gastric Cancer Cell Lines

**Figure 1:**
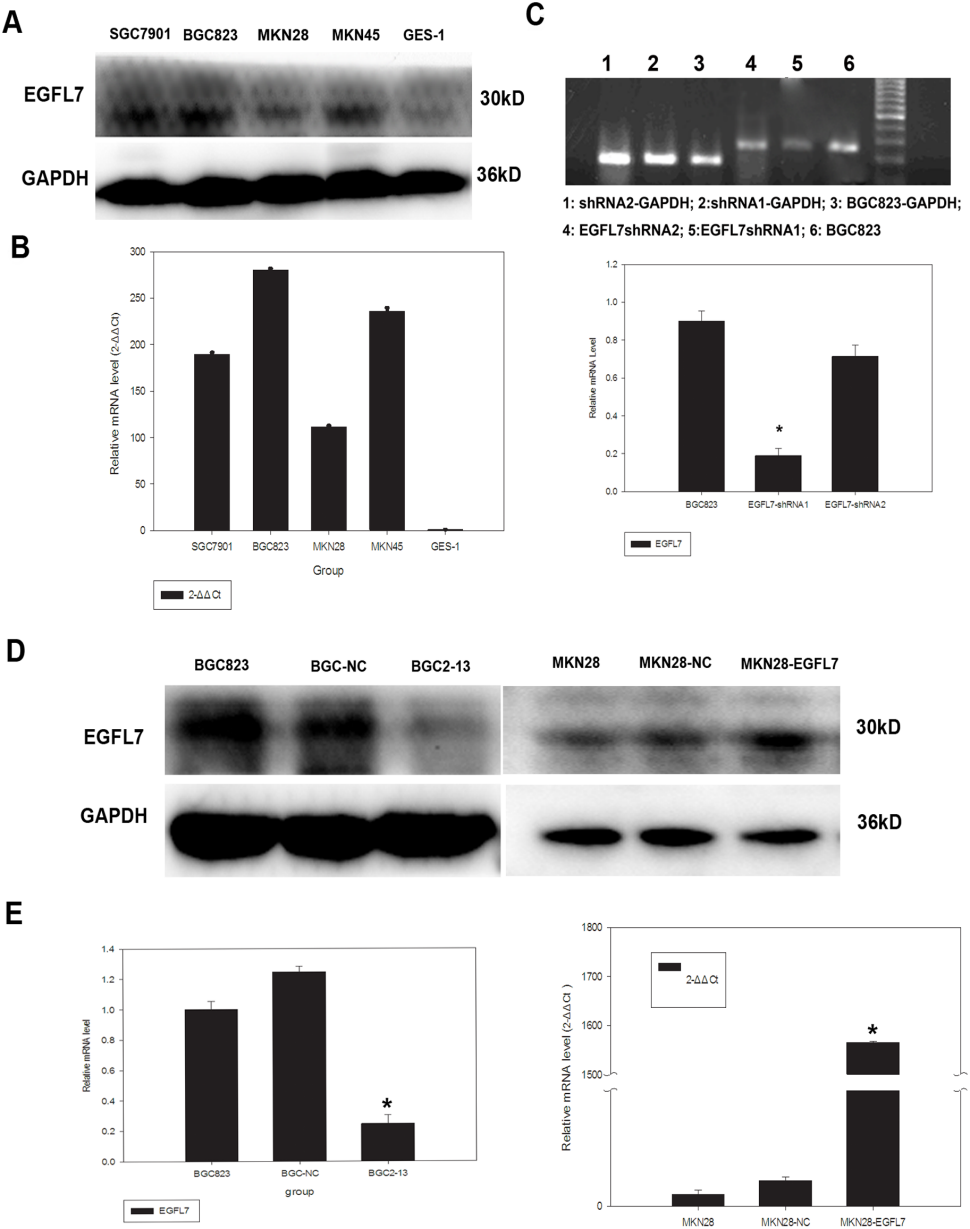
EGFL7 expression in native gastric cancer (GC) cell lines, normal gastric cells, and GC cell lines stably expressing an EGFL7 expression vector or targeted shRNA. (A) Expression levels of EGFL7 protein in the GC cell lines SGC7901, BGC823, MKN45, and MKN28, and the normal gastric cell line GES-1 were evaluated by Western blot. The GC cell lines showed higher EGFL7 protein expression levels than GES-1 cells, with BGC823 cells having the highest EGFL7 protein expression levels. (B) qRT-PCR revealed that BGC823 had the highest EGFL7 mRNA expression. Western blot and PCR experiments were performed in triplicate, and GAPDH was used as the internal control. (C) qRT-PCR showed that the shRNA1 sequence resulted in 75% inhibition of EGFL7 mRNA expression, whereas the shRNA2 sequence resulted in only 30% inhibition. (D) BGC823 cell lines stably transfected with pGPU6/GFP/Neo-EGFL7-shRNA1 or pGPU6/GFP/Neo-nonspecific-shRNA are designated BGC2-13 and BGC-NC, respectively. MKN28 cell lines transfected with pEX-2-EGFL7 or pEX-2-nonspecific are designated MKN28-EGFL7 and MKN28-NC, respectively. Expression of EGFL7 protein was markedly lower in BGC2-13 cells compared to BGC823 and BGC-NC cells, and markedly higher in MKN28-EGFL7 cells compared to MKN28 and MKN28-NC cells. (E) EGFL7 expression levels were also analyzed by qRT-PCR, and results confirmed Western blot data. GAPDH served as the internal control for both qRT-PCR and Western blot. Error bars represent the SD of triplicate experiments (*P<0.05).

Western blot and RT-PCR were performed to compare EGFL7 expression in the human GC cell lines SGC7901, BGC823, MKN45, and MKN28 to that in normal gastric mucosa epithelial GES-1 cells. Elevated EGFL7 levels were found in all four GC cell lines compared to GES-1, with BGC823 and MKN28 displaying the highest and lowest EGFL7 expression, respectively, both at the protein and mRNA levels (Figures 1A and 1B). Therefore, BGC823 and MKN28 were used in subsequent experiments to assess the effects of EGFL7 expression on proliferation, migration, and metastatic potential. We employed stable shRNA transfection to suppress EGFL7 expression in BGC823 cells and designed a human EGFL7 high-expression plasmid (pEX-2-EGFL7) to increase EGFL7 expression in MKN28 cells.

We constructed two shRNA plasmid vectors targeting EGFL7 mRNA and conducted qRT-PCR to assess the efficacy of these candidate shRNA sequences to suppress EGFL7 compared to nonspecific sequences. The shRNA1 and shRNA2 sequences achieved 75% and 30% inhibition of EGFL7, respectively (Figure 1C), so the more efficient shRNA1 was used for all subsequent experiments. The BGC823 lines stably transfected with pGPU6/GFP/Neo-EGFL7-shRNA1 or pGPU6/GFP/Neo-nonspecific-shRNA were designated BGC2-13 and BGC-NC, respectively. The MKN28 lines stably transfected with pEX-2-EGFL7 and pEX-2-nonspecific were designated MKN28-EGFL7 and MKN28-NC, respectively. The expression levels of EGFL7 protein and mRNA in G418-resistant clones were also evaluated by Western blot (Figure 1D) and real-time RT-PCR (Figure 1E). The results showed markedly decreased EGFL7 expression in BGC2-13 cells compared to BGC-NC cells and significantly elevated expression in MKN28-EGFL7 cells compared to MKN28-NC cells (all *P*<0.05). There were no significant differences in mRNA and protein expression between BGC-NC and BGC cells or between MKN28-NC and MKN28 cells (all *P*>0.05).

### EGFL7 Promoted GC Cell Invasion and Migration, but did not Affect GC Cell Proliferation

**Figure 2:**
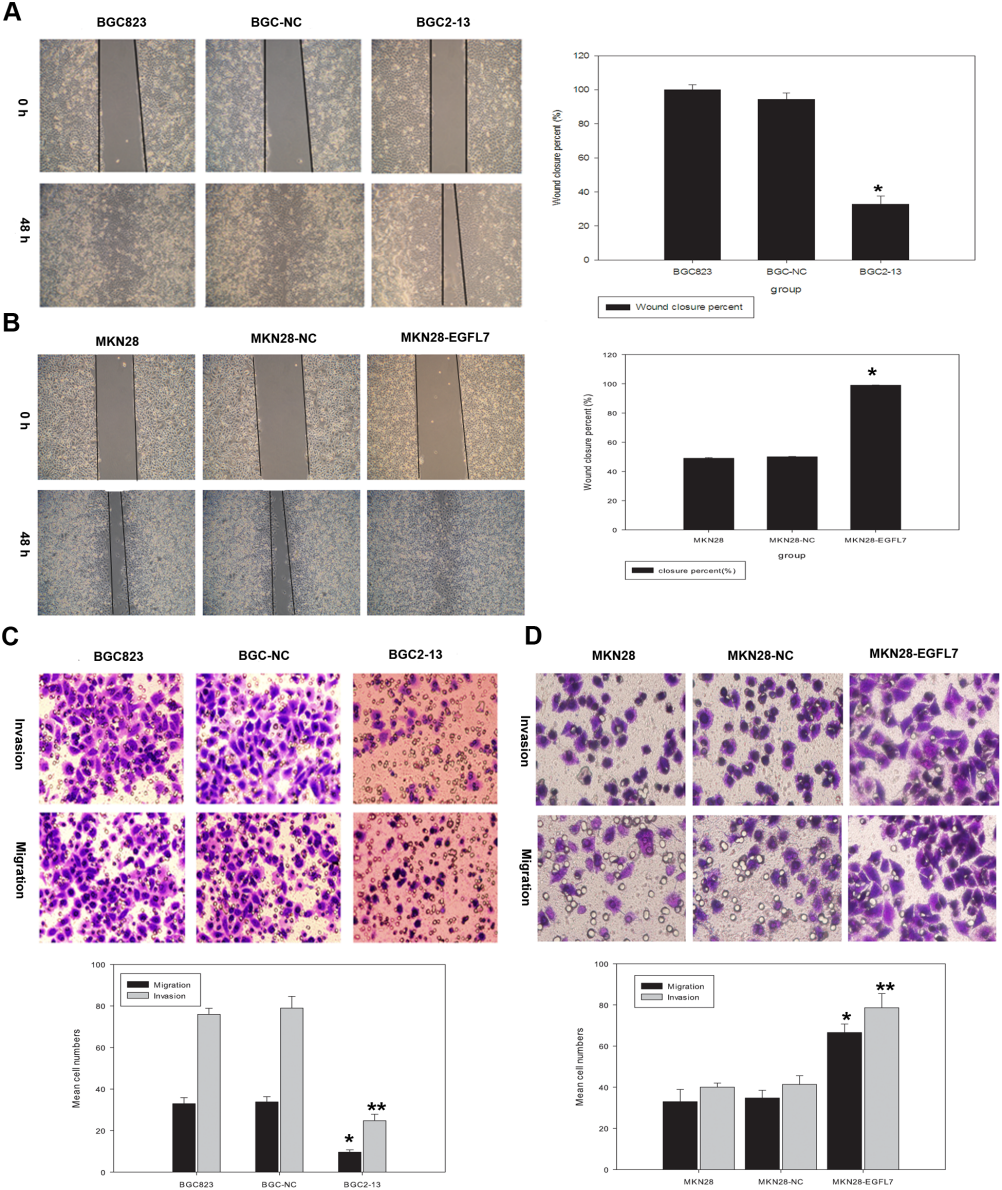
EGFL7 expression regulates GC cell migration and invasiveness. (A) Scratch wound healing was used to determine migration behavior in cell lines underexpressing or overexpressing EGFL7. A wound was generated and imaged at 0 and 48 h. Wound closure was significantly slower in (underexpressing) BGC2-13 cultures compared to BGC823 and BGC-NC cultures (32% *vs.* 100% and 98%, *P<0.05). (B) Wound closure was significantly faster in (overexpressing) MKN28-EGFL7 cultures compared to MKN28 and MKN28-NC cultures (99% *vs.* 49% and 50%, *P<0.05). (C) Invasive potential was assessed by the Matrigel invasion assay. Significantly fewer BGC2-13 cells passed through the Matrigel compared to BGC-NC and BGC823 cells (25±3.1 *vs.* 76±2.9 and 79±5.7, *P<0.05). For the transwell migration assay, cell lines were seeded in the upper chamber of the transwell. Significantly fewer BGC2-13 cells traveled through the transwell filter compared to BGC823 and BGC-NC cells (19±1.1 *vs.* 53±2.9 and 54±2.6, **P<0.05). (D) Significantly more MKN28-EGFL7 cells passed through the Matrigel to the lower side of the filter compared to MKN28-NC and MKN28 cells (79±6.19 *vs*. 40±2.00 and 41±4.22, *P<0.05). Similarly, significantly more MKN28-EGFL7 cells that traveled through the transwell filter compared to MKN28 and MKN28-NC cells (67±4.16 *vs*. 33±5.92 and 35±3.7, **P<0.05).

To investigate the role of EGFL7 in GC progression, we first examined whether EGFL7 silencing or overexpression altered the proliferative, invasive, and migratory capacities of GC cells. Scratch wound healing was used to measure migration of stably transfected cells. A wound was generated in GC cell monolayers by a single scratch and the number of cells in the scratch zone compared at 0 and 48 h. Closure of the wound was significantly slower in BGC2-13 cultures (underexpressing EGFL7) compared to native BGC823 and BGC-NC cultures (32% *vs.* 100% and 98%, both *P*<0.05) (Figure 2A). In contrast, closure was significantly faster in MKN28-EGFL7 cultures (overexpressing EGFL7) compared to MKN28 and MKN28-NC cultures (99% *vs.* 49% and 50%, both *P*<0.05) (Figure 2B). Moreover, wound closer at the some time post-scratch (48 h) was greater in high EGFL7-expressing wild type BGC823 cells compared to low-expressing wild type MKN28 cells, indicating that endogenous expression levels also impact migration.

**Figure 3:**
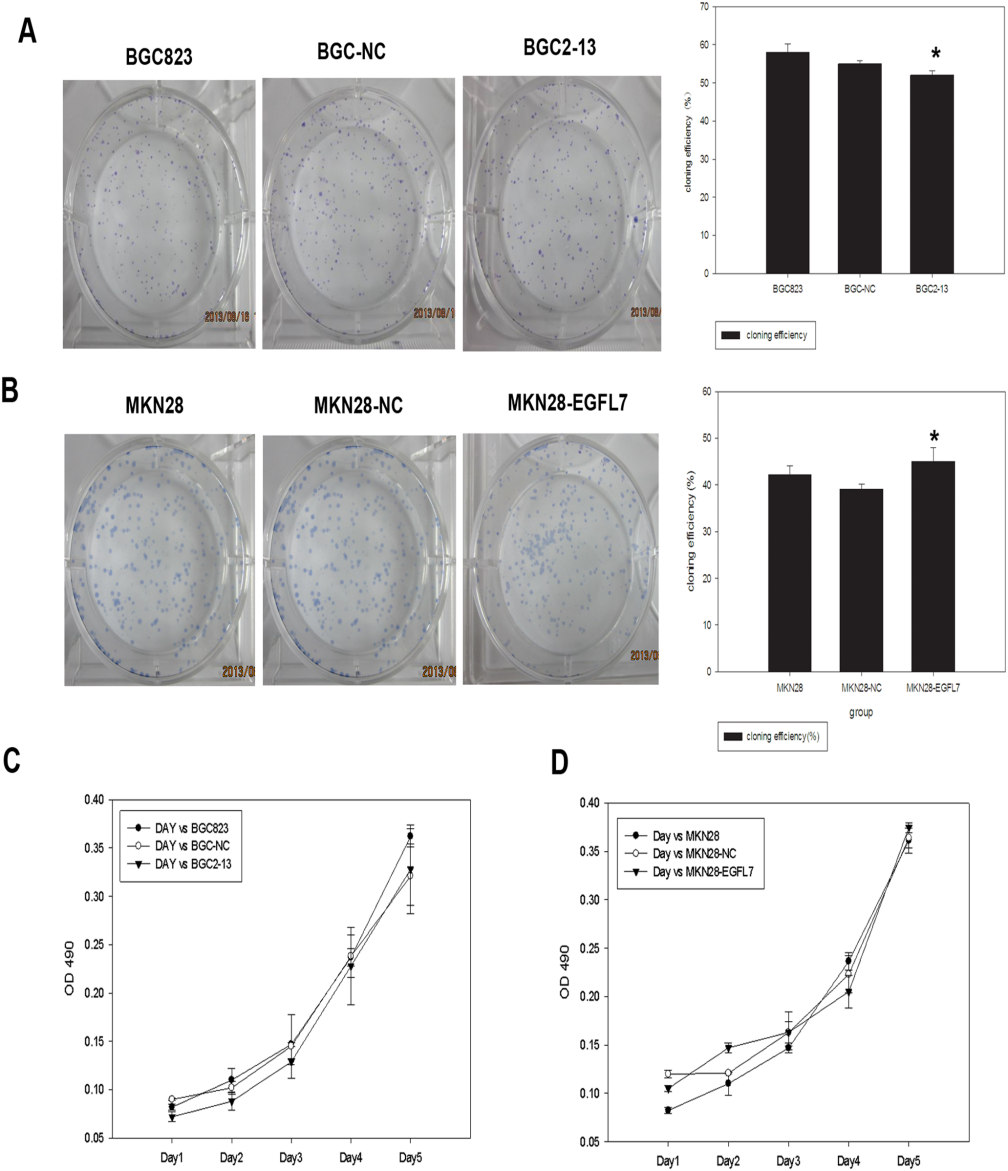
EGFL7 expression does not alter GC cell proliferation. (A) Effect of EGFL7 underexpression on cell proliferation as determined by the adherent plate colony formation assay. BGC823, BGC-NC, and BGC2-13 cells were plated at low density and colonies counted after 10 days. There was no significant difference in the number of colonies formed (52±1.1 *vs.* 58±2.2 and 55±0.8, *P>0.05) (mean ± SD from three independent experiments). (B) Effect of EGFL7 overexpression on cell proliferation. MKN28-EGFL7, MKN28-NC, and MKN28 cells were treated as above. There was no significant difference in the number of colonies formed (42±1.91 *vs.* 39±1.04 and 45±3.00, *P>0.05) (mean ± SD from three independent experiments). (C) Effect of EGFL7 underexpression on cell proliferation as measured by MTT assay. There was no significant difference in proliferation rate among BGC823, BGC-NC, and BGC2-13 cells. (D) Effect of EGFL7 overexpression on cell proliferation. There were also no significant differences in proliferation rates among the MKN28-EGFL7, MKN28-NC, and MKN28 cells. All data were expressed as mean ± SD and were obtained from three independent experiments.

Transwell assays were performed to confirm these findings and further investigate the effect of EGFL7 on the invasive potential of BGC823 cells, MKN28 cells, and the respective stable expression lines into Matrigel. There were significantly fewer EGFL7-underexpressing (BGC2-13) cells in the Matrigel compared to BGC-NC and BGC823 cells (25±3.1 *vs.* 76±2.9 and 79±5.7 cells/well; both *P*<0.05), indicating that EGFL7 underexpression significant reduced invasive capacity (Figure 2C). Similarly, the number of BGC2-13 cells reaching the lower Matrigel-free well in transwell migration assays was significantly reduced compared to BGC823 and BGC-NC cells (19±1.1 *vs.* 53±2.9 and 54±2.6, both *P*<0.05) (Figure 2C). In contrast, EGFL7 overexpression in MKN28 cells significantly enhanced both invasion into Matrigel (MKN28-EGFL7: 79±6.19 cells/well, MKN28-NC: 40±2.00 cells/well, MKN28: 41±4.22; both *P*<0.05; Figure 2D) and migration (MKN28-EGFL7: 67±4.16, MKN28-NC: 33±5.92, MKN28: 35±3.7; both *P*<0.05; Figure 2D). We also assessed the effect of EGFL7 expression on proliferation rate by colony formation (Figures 3A and 3B) and MTT (Figures 3C and 3D) assays, but found no significant differences between cell lines.

### EGFL7 Protects GC Cells from Anoikis in Suspension Culture

**Figure 4:**
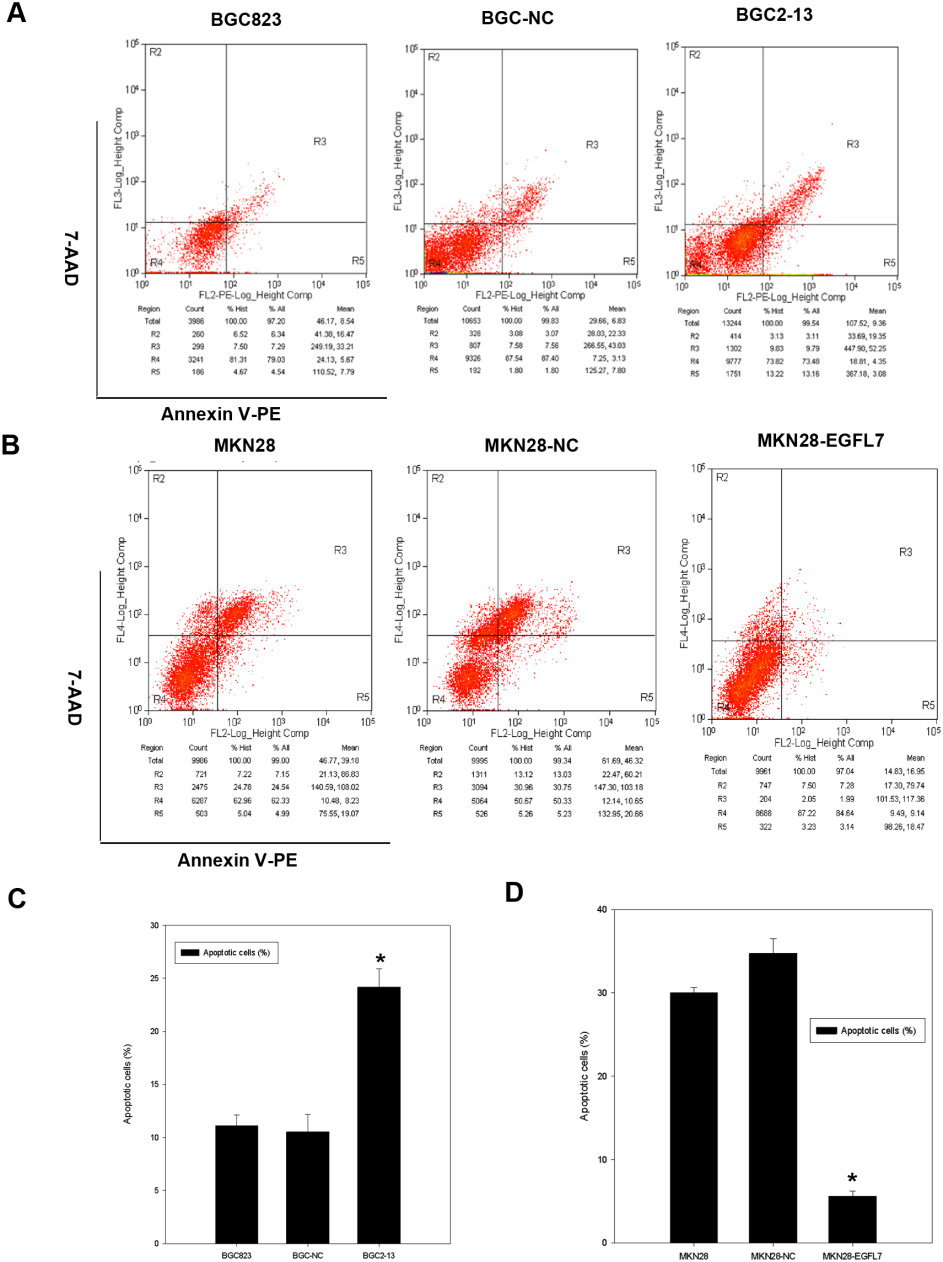
EGFL7 induces anoikis resistance of GC cells in suspension culture. (A) Effect of EGFL7 underexpression on anoikis resistance. Representative cytograms from flow cytometry analysis of apoptotic cells revealed by Annexin V-PE/7-AAD staining of BGC823, BGC-NC, and BGC2-13 cells after 24 h in suspension culture. A greater percentage of BGC2-13 cells (22.95±1.72%) were apoptotic compared to BGC823 (11.83% ±0.99%) and BGC-NC cells (9.36% ±1.65%). (B) Effect of EGFL7 overexpression on anoikis resistance. Representative cytograms from flow cytometry analysis of apoptotic cells revealed by Annexin V-PE/7-AAD staining of MKN28-EGFL7, MKN28-NC, and MKN28 cells after 24 h of suspension culture. The number of apoptotic MKN28-EGFL7 cells (5.13% ±0.65%) was lower than the number of apoptotic MKN28 (29.53% ±0.68%) and MKN28-NC cells (35.98% ±1.77%). (C) and (D) Flow cytometry results plotted as the mean ± SD of triplicate experiments. *P<0.05 considered significant. All experiments were performed in triplicate and repeated at least three times.

Many cell types undergo programmed cell death when deprived of an adherent substrate, termed anoikis. Gastric cancer cells were maintained in suspension culture by coating the culture plates with poly-HEMA and anoikis measured after 24 h by flow cytometry measurement of annexin V-PE/7-AAD staining. A significantly higher percentage of EGFL7-underexpressing BGC2-13 cells were apoptotic compared to BGC823 and BGC-NC cells (22.95% ±1.72% *vs.* 11.83% ±0.99% and 9.36% ±1.65%, both *P*<0.05) (Figures 4A and 4C), while a significantly lower percentage of EGFL7-overexpressing MKN28-EGFL7 cells were apoptotic compared to both MKN28 and MKN28-NC cells (5.13% ±0.65% *vs.* 29.53% ±0.68% and 35.98% ±1.77%, both *P*<0.05) (Figures 4B and 4D). Thus, EGFL7 expression confers resistance to anoikis as required for GC metastasis to distal sites.

### Knockdown or Overexpression of EGFL7 Modulates the Growth and Metastasis of GC Xenograft Tumors in Nude Mice

**Figure 5:**
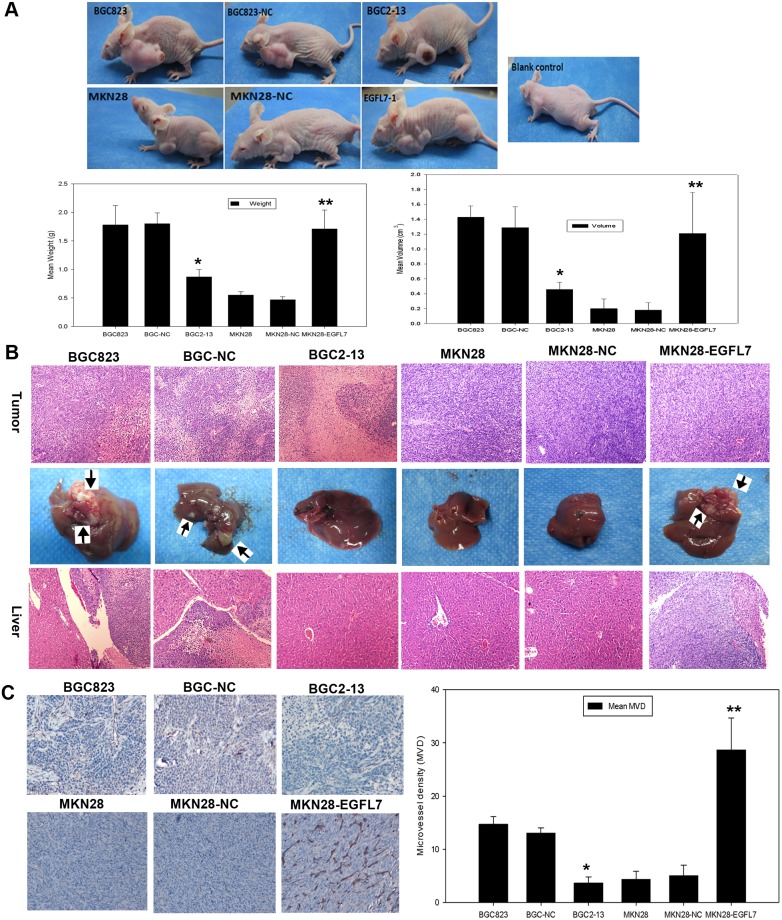
EGFL7 modulates the growth and metastasis of GC xenograft tumors in nude mice. (A) Subcutaneous xenograft tumors were significantly smaller in BGC2-13 cell-injected mice compared to BGC823 cell- and BGC-NC cell-injected mice, while MKN28-EGFL7 cell-injected mice exhibited significantly larger tumors than MKN28 cell- and MKN28-NC cell-injected mice. Tumor volume was calculated according to tumor volume (mm^3^) = 0.5×length×width^2^ (*P<0.05, **P<0.01). (B) Subcutaneous xenograft tumors were analyzed by H&E staining. Liver metastasis was observed in mice injected with high EGFL7-expressing cells (BGC823, BGC-NC, and MKN28-EGFL7). Black arrow, metastatic cancer cells in the liver tissues of nude mice (H&E staining, original magnification×200). (C) Average MVD of tumors was lower in the BGC2-13 group compared to the BGC823 and BGC-NC groups (4±1.2 *vs.* 15±2 and 13±1, *P<0.05), while the average MVD of tumors was higher in the MKN28-EGFL7 group than the MKN28 and MKN28-NC groups (28.7±6.02 *vs.* 4.3±1.53 and 5.0±2.0, **P<0.05).

To validate these *in vitro* studies showing enhanced migration and invasion, BGC823, BGC-NC, BGC2-13, MKN28, MKN28-NC and MKN28-EGFL7 cells were implanted subcutaneously into the left upper extremity of nude mice (5 mice/group). Mean volume and tumor weight were smaller in the BGC2-13 group than the BGC823 and BGC-NC group 4 weeks after implantation (Figure 5A). Conversely, tumors were larger in mice injected with MKN28-EGFL7 cells compared to tumors in mice injected with MKN28 or MKN28-NC cells (Figure 5A). Ischemic necrosis was observed on the surface of tumors in the BGC2-13 group, possibly reflecting a lack of angiogenesis. Indeed, microvessel density (MVD) as detected by CD34 immunohistochemistry was lower in tumors arising from BGC2-13 cell injection compared to those arising from BGC823 and BGC-NC injection (4±1.2 *vs.* 15±2 and 13±1, both *P*<0.05) (Figure 5C). In contrast, the average MVD of the tumors from MKN28-EGFL7 cell injection was significantly higher than in tumors arising from MKN28 and MKN28-NC cell injection (28.7±6.02 *vs.* 4.3±1.53 and 5.0±2.0, both *P*<0.05) (Figure 5C). These findings indicated that EGFL7 has a pivotal role in GC angiogenesis.

*In vitro* migration, invasion, and anoikis assays suggest that cells overexpressing EGFL7 (MKN28-EGFL7) should also show enhance metastasis *in vivo* whilst underexpressing cells (BGC2-13) should exhibit lower metastasis. To investigate metastasis of these different cell lines *in vivo*, we analyzed mouse livers histologically by H&E staining. Consistent with *in vitro* results, we observed more metastatic cancer cells in liver tissues from mice injected with MKN28-EGFL7, BGC823, or BGC-NC cells compared to mice injected with BGC2-13, MKN28, or MKN28-NC cells (Figure 5B) indicating that overexpression (whether via transfection or endogenous) promotes liver metastasis of GC cells.

### EGFL7 Promotes EMT of GC Cells through AKT Phosphorylation

**Figure 6:**
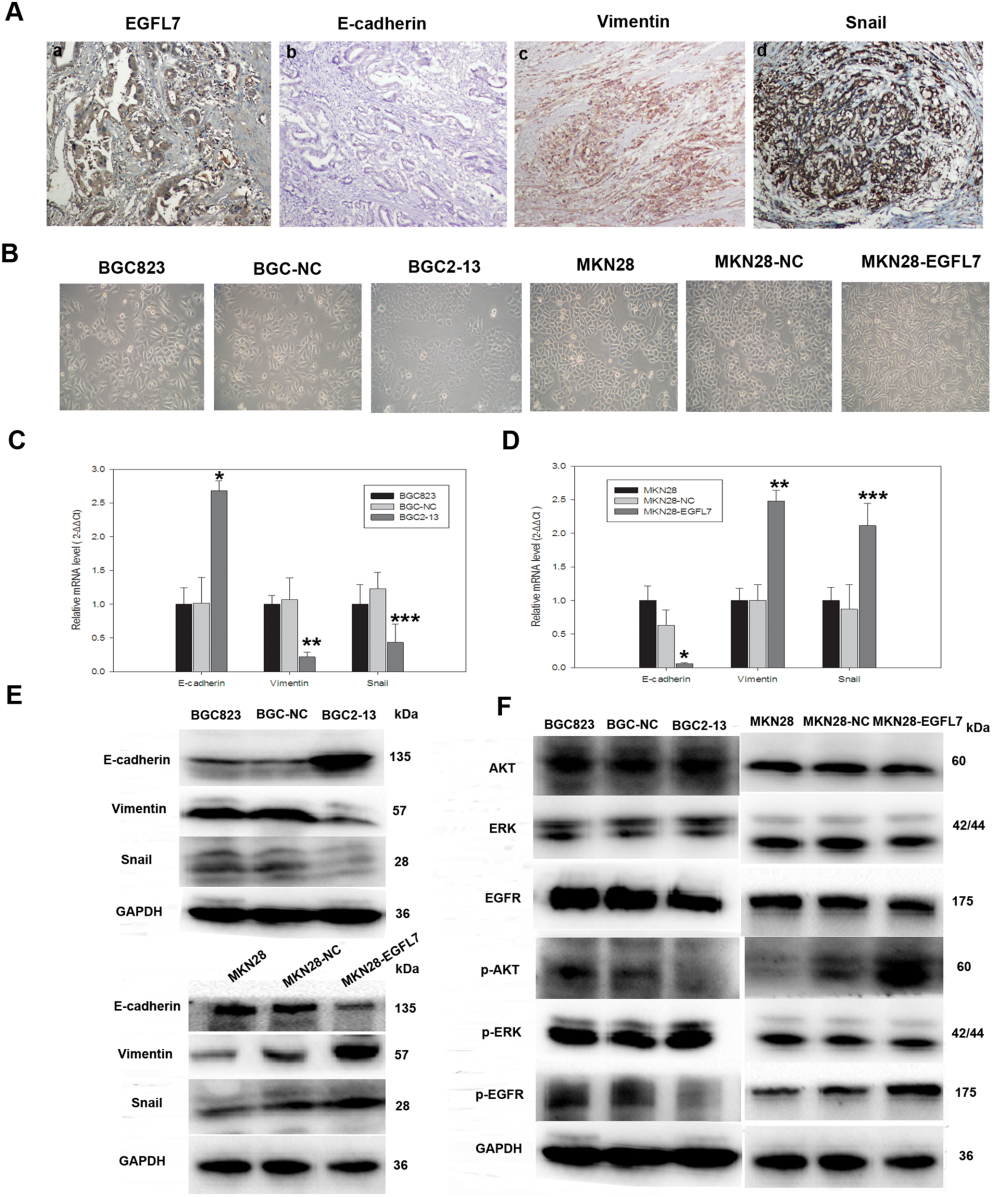
EGFL7 promotes Epithelial−Mesenchymal transition (EMT) of GC cells through the EGFR–AKT signaling pathway. (A) Immunohistochemistry showing EGFL7, E-cadherin, vimentin, and Snail expression in gastric carcinoma tissues. (B) Cellular morphology of EGFL7-underexpressing cells (BGC2-13, MKN28, and MKN28-NC) was distinct from that of EGFL7-overexpressed cells (BGC823, BGCNC, and MKN28-EGFL7). BGC823, BGCNC, and MKN28-EGFL7 cells exhibited loss of intercellular contacts and typical spindle-shaped mesenchymal cell morphology, whereas BGC2-13, MKN28, and MKN28-NC cells exhibited an epithelial cell-like morphology with small cell size and cobblestone-like shape with tightly arranged intercellular contacts. (C) and (D) Expression levels of EMT-related molecules in cell lines analyzed by qRT-PCR (*P<0.05, **P<0.05, ***P<0.05). (E) Western blot was used to confirm changes in expression of EMT-related molecules. qRT-PCR and Western blot results showed higher E-cadherin mRNA and protein expression levels in BGC2-13 cells and lower mRNA and protein expression levels of the mesenchymal markers vimentin and Snail. GAPDH served as an internal control for qRT-PCR reactions and Western blot. Error bars represent SD of triplicate experiments (*P<0.05, **P<0.05, ***P<0.05 compared to BGC823 and BGC-NC cells). Conversely, qRT-PCR and Western blot results showed lower E-cadherin mRNA and protein expression levels in MKN28-EGFL7 cells and higher mRNA and protein expression levels of mesenchymal markers vimentin and Snail. Error bars represent SD of triplicate experiments (*P<0.05, **P<0.05, ***P<0.05, compared to MKN28 and MKN28-NC cells). (F) Western blots showing both total and phosphorylated levels of EGFR, ERK1/2, and AKT in BGC823, BGC-NC, BGC2-13, MKN28-EGFL7, MKN28-NC, and MKN28 cells. Phosphorylated EGFR and AKT levels (pEGFR and pAKT) were significantly lower in BGC2-13 cells than in BGC823 and BGC-NC cells, while pEGFR and pAKT expression levels were significantly higher in MKN28-EGFL7 cells compared to MKN28 and MKN28-NC cells. Total EGFR and AKT levels did not differ significantly among cell lines. Neither total ERK1/2 nor pERK1/2 differed significantly. Western blots were performed in triplicate.

We next explored the mechanisms through which EGFL7 promotes GC EMT and metastasis. The expression levels and subcellular location of EGFL7 and the three EMT-associated markers, vimentin, Snail, and E-cadherin, in GC tissue samples are depicted in Figure 6A and Table 1. EGFL7 was mainly located in the cytoplasm, and positive expression (Figure 6A–a) was detected in 96.2% of gastric cancer samples (76/79). E-cadherin was mainly confined to cell membranes (Figure 6A–b), while Snail was observed in nuclei of cancer cells (Figure 6A–d), and vimentin was expressed in the cytoplasm (Figure 6A–c). To determine whether EGFL7 expression is associated with the levels of these other EMT-related molecules, correlation analyses were performed. As shown in Table 1, EGFL7 expression was positively correlated with expression levels of the mesenchymal markers vimentin (*r* = 0.620, *P*<0.05) and Snail (*r* = 0.492, *P*<0.05) and negatively correlated with expression of the epithelial marker E-cadherin (*r* = –0.304, *P*<0.05).

**Table 1:** Relationship between EGFL7 and EMT-Related Molecule Protein Expressions in Gastric Cancer.

Characteristics	EGFL7, N (%)			*r*	*P* value	
	No expression	Weak expression	Strong expression			
	(n = 3)	(n = 17)	(n = 59)			
E-cadherin				–0.304	0.009*	
Negative	0	3(17.6%)	10(16.9%)			
Weak	0	3(17.6%)	32(54.2%)			
Strong	3(100%)	11(64.8%)	17(28.9%)			
Vimentin				0.620	<0.001*	
Negative	1(33.3%)	2(11.8%)	1(1.7%)			
Weak	1(33.3%)	10(58.8%)	4(6.8%)			
Strong	1(33.3%)	5(29.4%)	54(91.5%)			
Snai1				0.492	<0.001*	
Negative	0	3(17.6%)	1(1.7%)			
Weak	0	7(41.2%)	2(3.4%)			
Strong	3(100%)	7(41.2%)	56(94.9%)			

*Note*: *r* is Spearman’s rank correlation coefficient; two-tailed significances,

*P<0.05.

Cells expressing high levels of EGFL7 (BGC823, BGC-NC, and MKN28-EGFL7 lines) exhibited a spindle-shaped morphology typical of mesenchymal cells, whereas cells expressing lower levels (BGC2-13, MKN28, and MKN28-NC lines), were smaller, cobblestone-shaped, and tightly arranged with intercellular contacts typical of epithelial cells (Figure 6B). We then compared the basal expression levels of E-cadherin and vimentin in all stably transfected cells by qRT-PCR and Western blot. As predicted, both E-adherin mRNA and protein expression levels were higher in BGC2-13 cells (underexpressing EGFL7) whereas vimentin mRNA and protein expression levels were lower compared to BGC823 and BGC-NC cells (Figures 6C and 6E). In addition, increased EGFL7 expression in MKN28-EGFL7 cells was associated with enhanced expression of vimentin and reduced expression of E-cadherin at both mRNA and protein levels (Figures 6D and 6E). These findings suggest that EGFL7 expression is associated with EMT. Conversely, EGFL7 knockdown results in EMT reversal, causing mesenchymal to epithelial transition (MET).

**Figure 7:**
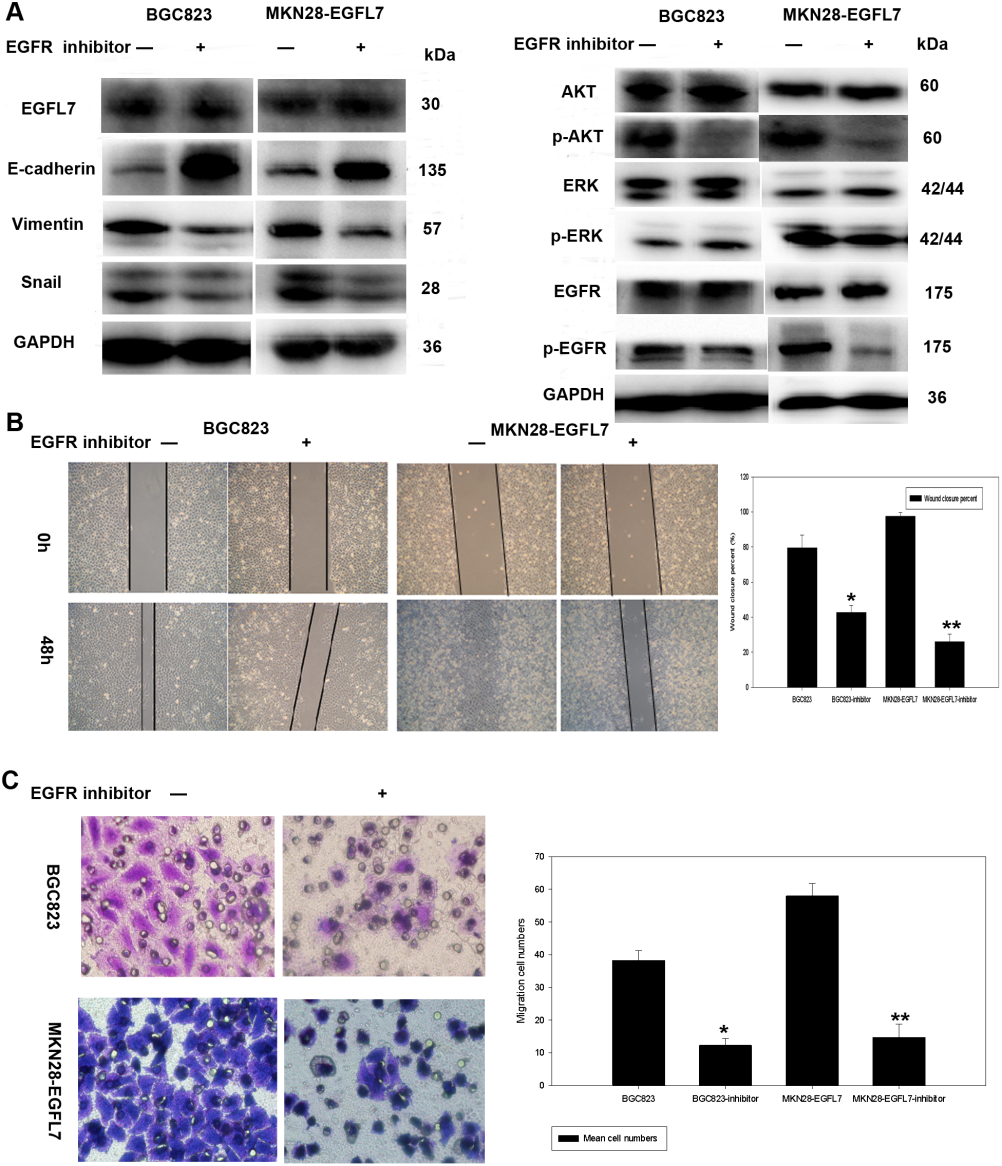
An EGFR tyrosine kinase inhibitor (Tyrphostin AG1478) blocks the effect of EGFL7 on EGFR phosphorylation, AKT phosphorylation, and cell motility. (A) Western blots showing that expression of EMT-related proteins changed significantly in BGC823 and MKN28-EGFL7 cells after treatment with the EGFR inhibitor Tyrphostin AG1478 (20 µM) for 1 h compared to vehicle (0.25% DMSO). E-cadherin protein expression levels increased in BGC823 and MKN28-EGFL7 cells after Tyrphostin AG1478 treatment, while expression levels of the mesenchymal marker proteins vimentin and Snail decreased. pEGFR and pAKT expression levels were significantly lower in BGC823 and MKN28-EGFL7 cells treated with Tyrphostin AG1478 than in BGC823 and MKN28-EGFL7 cells treated with 0.25% DMSO. No changes in EGFL7 protein expression levels were observed in BGC823 and MKN28-EGFL7 cells after treatment with Tyrphostin AG1478. (B) Migration of BGC823 and MKN28-EGFL7 cells was inhibited by Tyrphostin AG1478 treatment in the scratch wound assay (79% *vs.* 49%, *P<0.05; 97% *vs.* 26%, **P<0.05). (C) Tyrphostin AG1478 also reduced migration in the transwell assay (37±3.9 *vs.* 18±1.5, *P<0.05; 58±3.7 *vs.* 15±4.0, **P<0.05; vehicle-treated *vs.* Tyrphostin AG1478-treated).

To investigate the molecular signaling pathways involved in EMT regulation by EGFL7, we first used qRT-PCR to estimate the mRNA levels of Snail, the transcriptional repressor of E-cadherin (Figure 6). Knockdown of EGFL7 (BGC2-13 cells) significantly decreased Snail expression (Figure 6C) while EGFL7 overexpression (MKN28-EGFL7 cells) increased Snail levels (Figure 6D). Dysregulation of epithelial growth factor signaling through AKT and ERK is a critical event in tumorigenesis. We thus explored the effects of EGFL7 on phospho-activation of EGFR, AKT, and ERK. Phosphorylation of both EGFR and AKT was significantly lower in BGC2-13 cells compared to BGC823 and BGC-NC cells (Figure 6F). Conversely, phosphorylation of both EGFR and AKT was significantly higher in MKN28-EGFL7 cells compared to MKN28 and MKN28-NC cells (Figure 6F). However, no differences in ERK phosphorylation were observed among the various cell lines (Figure 6F). A previous study reported that the effects of EGFL7 are mediated by the EGFR signaling pathway, which is critical for controlling cancer cell motility [10]. Therefore, we examined whether EGFR-induced AKT activation increases EMT and metastasis in GC cells. EGFR phosphorylation was blocked by Tyrphostin AG1478 (a tyrosine kinase inhibitor selective for EGFR previously shown to block EGFR activation *in vivo*; Cell signaling Technology, Inc., USA) in cells overexpressing EGFL7 (BGC823 and MKN28-EGFL7 lines). No change in EGFL7 protein expression was observed in BGC823 and MKN28-EGFL7 cells after treatment with Tyrphostin AG1478 (Figure 7A) compared to cells treated with vehicle (0.25% DMSO), indicating that EGFL7 acts upstream of EGFR. EGFR and AKT phosphorylation levels in BGC823 and MKN28-EGFL7 cells decreased significantly following Tyrphostin AG1478 treatment (Figure 7A), and this was associated with reduced expression of EMT-related molecules and reversal of EMT (Figure 7A). Moreover, transwell (Figure 7C) and scratch wound (Figure 7B) assays both showed that Tyrphostin AG1478 blocked the elevated cell motility of BGC823 and MKN28-EGFL7 lines. These findings strongly suggest that EGFL7 promotes EMT and metastasis through EGFR-mediated AKT phosphorylation.

## Discussion

Invasion and metastasis are major obstacles to the curative treatment of malignancy and the most frequent cause of cancer-related death. In our previous study, high expression of EGFL7 was found to correlate positively with the invasion and metastasis of gastric carcinoma [11]. Here, we explored the functional effects of EGFL7 in GC cells and the associated molecular mechanisms. We demonstrate that EGFL7 overexpression enhances GC cell migration and invasive capacity, reduces anoikis, and promotes EMT (with concomitant reduction in E-cadherin expression), characteristics that could facilitate metastasis. Indeed, human GC cells overexpressing EGFL7 were more aggressive and more likely to metastasize to the liver from subcutaneous mouse xenografts. While the signaling mechanisms responsible for these effects are not completely clear, we show that EGFL7 activates the mitogenic EGFR−AKT pathway and increases Snail expression, a major transcriptional repressor involved in EMT. Significantly, EGFL7 underexpression reduced migration, invasion, and EGFR−AKT phospho-activation *in vitro* and metastasis in mice. Inhibition of the EGFR tyrosine kinase also blocked enhanced migration in EGFL7-overexpressing cells. These results strongly suggest that EGFL7 increases tumor aggression at least in part by EGFR−AKT signaling and underscores EGFL7 as a promising candidate for therapeutic intervention.

Cancer metastasis is a multi-step cascade that starts with EMT, dissociation from the primary tumor, and distal invasion [23]. Invasion into healthy tissue is thus a barrier to the progression of metastatic disease. Therefore, we assessed the invasive capacity of GC cells expressing variable levels of EGFL7. Lines with higher expression showed a more aggressive phenotype, including greater invasion into matrigel and migration into scratch wounds and across transwell barriers. Enhanced aggression of EGFL7-overexpressing GC cells was also observed in mouse xenografts. Conversely, EGFL7 knockdown decreased GC metastasis in liver. Overexpression of EGFL7 also increased tumor size *in vivo*, in agreement with previous studies of hepatic carcinoma [10]. However, while knockdown of EGFL7 expression by a targeted shRNA reduced tumor size *in vivo*, this treatment had no effect on GC cell proliferation *in vitro*. The surface of tumors from low EGFL7-expressing GC cells exhibited ischemic necrosis, suggesting that EGFL7 knockdown may reduce tumor size not by reducing proliferation but by suppressing angiogenesis. Indeed, the average MVD was lower in xenograft tumors arising from EGFL7-underexpressing BGC2-13 cells compared to tumors arising from injection of high EGFL7-expressing BGC823 and BGC-NC cells, consistent with previous reports of angiogenesis inhibition by EGFL7 suppression [24], [25]. Conversely, the average MVD was higher in tumors from EGFL7-overexpressing MKN28-EGFL7 cells, suggesting that EGFL7 promotes tumor growth through angiogenesis rather than direct effects on proliferation.

Anoikis, a form of programmed cell death in response to detachment of cells from other cells or the extracellular matrix, represents another physiological barrier to metastasis [26]. A number of studies have demonstrated that anoikis inhibits metastasis of various solid tumors [27], [28], while resistance against anoikis is considered a prerequisite for cancer cell survival in the circulation prior to distal invasion (metastasis) [28]. The anoikis rate in BGC2-13 cells was increased by EGFL7 knockdown, consistent with the lower metastasis capacity of this line in mice. Alternatively, the relative anoikis resistance of MKN28-EGFL7 cells may have enhanced metastatic capacity.

Tumor metastasis also requires EMT [29]−[31]. We observed that EGFL7 knockdown in normally high EGFL7-expressing BGC823 cells (a poorly differentiated adenocarcinoma line) resulted in a change from the typical spindle-shaped mesenchymal cell morphology to an epithelial cell-like morphology (a sign of MET). Conversely, EGFL7 overexpression in the normally low EGFL7-expressing MKN28 line resulted in transition from a typical epithelial cell-like morphology to a spindle-shaped mesenchymal cell morphology (EMT). For metastasis, carcinoma cells must temporarily lose defining features, such as cell–cell adhesion, epithelial tight junction, and desmosomes [14], [32], [33]. Epithelial cells are normally polarized and tightly connected to one another by intercellular junctions that prevent motility. By contrast, mesenchymal cells do not establish stable intercellular contacts and have greater locomotor capacity [32], [33]. The EMT has been shown to contribute to tumor formation and metastasis of GC [13], [34], [35]. Recent studies have also revealed that the typical EMT profile is correlated with tumor grade and metastasis of GC [34], [36], [37]. Immunohistochemical analysis of three EMT-associated markers in 79 GC patients revealed that EGFL7 expression was positively correlated with expression of the mesenchymal markers vimentin and Snail, and negatively correlated with expression of the cell adhesion protein E-cadherin. Furthermore, E-cadherin expression was higher in EGFL7-underexpressing BGC2-13 cells compared to native BCG823 cells (which express relatively high levels of endogenous EGFL7), whereas expression of the mesenchymal marker vimentin was lower. Conversely, EGFL7-overexpressing MKN28-EGFL7 cells exhibited low E-cadherin expression and high Snail and vimentin expression compared to the control lines. Thus, EGFL7 expression was associated with EMT, and suppression of EGFL7 expression resulted in MET, possibly explaining the low invasive, migratory, and metastatic capacities of EGFL7-underexpressing cells.

Activation of AKT promotes EMT of colorectal cancer cells [38], and accumulating evidence also supports a critical role for EGFR, an upstream activator of AKT, in promoting EMT-like phenotypes in mammary epithelial cells, hepatocytes [39], [40], and lung epithelial cells [41]. Furthermore, a recent study demonstrated that EMT is required for EGF-induced large gastric cancer (LGC) and ovarian cancer cell migration and invasion, and that EGF-induced EMT involves activation of the ERK1/2 and PI3K/AKT pathways with subsequent induction of Snail, Slug, and ZEB1 expression [18]. Notably, AKT phospho-activation downregulates E-cadherin expression and promotes EMT-like transition and invasiveness in carcinoma cells by inducing Snail [42]. These studies suggest that EGFL7 promotes EMT by facilitating AKT phosphorylation, which negatively regulates the transcription of E-cadherin by activating Snail. Cell lines with low EGFL7 expression also exhibited low EGFR and AKT phosphorylation compared to EGFL7-overexpressing lines. In addition, EMT confers anoikis resistance in melanoma and colon cancer cells [43]. Downregulation of epithelial markers and concomitant upregulation of mesenchymal markers is an indicator of EMT, and several EMT-associated proteins, such as TrkB, casein kinase 3, and N-cadherin, are also associated with anoikis resistance [29], [44], [45]. Additionally, the phosphoinositide 3-kinase (PI3K)/AKT pathway has been identified as an important downstream target of treatments that enhance the anoikis resistance of tumor cells [46]. Additional studies are necessary to determine whether these proteins are also involved in anoikis resistance in GC cells overexpressing EGFL7.

SNAIL, SLUG, Twist1, and Twist2 are repressors of E-cadherin during EMT-like transition [47]. The zinc-finger factor Snai1, a member of the Snail superfamily of transcriptional repressors, has an important role in E-cadherin downregulation and in the induction of EMT during embryogenesis, cancer progression, and metastasis [48], [49]. Wu et al. concluded that EGFL7 expression is controlled by EGFR signaling, a pathway also critical in controlling cancer cell motility [10]. Treatment of BGC823 cells and MKN28-EGFL7 cells with an EGFR inhibitor (Tyrphostin AG1478) blocked EGFR and AKT phosphorylation as well as expression of EMT-related proteins. Tyrphostin AG1478 also reduced cell migration, suggesting that EGFL7 may promote cell migration and invasion by activating EMT through the EGFR signaling pathway.

The Notch signaling pathway has also been implicated in EMT induction [50]−[52]. For example, the Notch-1 receptor ligand Jagged-1 induced mesenchymal transformation of endothelial cells [53]. EGFL7 reduced neural stem cell (NSC) self-renewal by acting as a Jagged-1 antagonist and inhibitor of Notch receptor-mediated signaling [54]. As EGFL7 is a soluble protein, it cannot create the mechanical force necessary to effectively separate the Notch heterodimer. Rather, EGFL7 competes with Notch ligands of the Jagged type, perhaps by binding to an overlapping region on Notch-1 [55], [56]. Thus, EGFL7 may also induce EMT by inhibiting Notch signaling. Multiple oncogenic pathways, such as NF-κB, AKT, Sonic hedgehog (Shh), mammalian target of rapamycin (mTOR), Ras, Wnt, estrogen receptor (ER), androgen receptor (AR), EGFR, and platelet-derived growth factor (PDGF), have been reported to interact with the Notch signaling pathway [57]. Moreover, Notch target genes include nuclear factor-kappa B (NF-κB), AKT, and vascular endothelial growth factor (VEGF) [58]−[60]. Thus, cross-talk between Notch and other signaling pathways may regulate tumor aggressiveness, including interactions with EGFR, which has been proposed as an upstream regulator of Notch through a non-autonomous cellular mechanism [61]. Therefore, we suggest that EGFL7 acts through EGFR-mediated regulation of Notch signaling to induce EMT, although confirmation requires further study.

In conclusion, our data suggest that EGFL7 is an important regulator of GC cell metastasis. EGFL7 is overexpressed in GC and this overexpression promotes gastric tumorigenesis and metastasis. Moreover, we have demonstrated for the first time that the EMT-associated transcription suppressor Snail is regulated by EGFL7 through the EGFR−AKT pathway.

## Supporting Information

Checklist S1 (DOC)
