# Supplementary material for: Epidermal Growth Factor-Like Domain-Containing Protein 7 (EGFL7) Enhances EGF Receptor−AKT Signaling, Epithelial−Mesenchymal Transition, and Metastasis of Gastric Cancer Cells
Source: PLoS One. 2014 Jun 19;9(6):e99922. doi: 10.1371/journal.pone.0099922 (PMC4063792; doi:10.1371/journal.pone.0099922)
Supplement: Checklist S1 — (DOC) [file pone.0099922.s001.doc]

The ARRIVE Guidelines Checklist

Animal Research: Reporting In Vivo Experiments

**Title:**

**Increased Metastasis Induced by EGFL7 in Xenograft Cancer Model of Gastric Cancer**

**Abstract:**

BACKGROUND AND PURPOSE: As a potential biomarker of human epithelial tumors, epidermal growth factor-like domain 7 (EGFL7) is significantly upregulated in human epithelial tumor tissues. Previous studies proved that high expression of EGFL7 promotes the infiltration and metastasis of gastric carcinoma. In this study, we showed that suppressing EGFL7 expression can prevent cell invasion and migration. We further studied the effect of EGFL7 in gastric cancer tumorigenesis and metastasis in xenograft models.

EXPERIMENTAL APPROACH: Twenty female Balb/c nude mice aged 4 to 6 weeks were purchased from the Animal Experimental Centre of the Third Xiangya Hospital, Central South University. They were divided randomly into four groups of five. BGC2-13, BGC-NC, and BGC823 cells were detached and resuspended in PBS. Then, 0.2 ml of each suspension containing 1×10^8^ cells was injected into the left upper extremity of each mouse, using PBS as the control. Tumor growth was evaluated by measuring tumor diameters with a Vernier caliper every 3 d. Tumor volume (TV) was calculated according to the formula: TV (cm^3^) = d^2^ ×D/2, where d and D represent the shortest and the longest diameters, respectively. The mice were executed by method of cervical dislocation after 4-5 weeks, and tumors were removed and weighed. All organs were examined for metastasis. The removed tumors were fixed in 10% formalin, embedded in paraffin, and then cut into 4 μm-thick sections. MVD expression was determined by immunohistochemistry.

KEY RESULTS: We have demonstrated that the tumors in the BGC2-13 group were smaller than those in the BGC823 and BGC-NC groups, and we also have abserved that more metastatic cancer cells in live tissues of BGC823 and BGC-NC groups than in live tissues of BGC2-13 group. In addition, MVD was calculated in tumors of the three groups, and the results showed that the average MVD of the tumors in the BGC2-13 group was lower than that in the BGC823 and BGC-NC groups.

CONCLUSIONS AND IMPLICATIONS: EGFL7 was found to be crucial in the regulation of gastric cancer metastasis.

**Background**

Gastric cancer (GC) remains the fourth most common malignant tumor worldwide and the second leading cause of cancer-related mortality [1]. The significant progress in the systemic treatment of GC has greatly increased the overall survival of GC patients; however, the overall five-year survival rate of GC patients remains low because of relapse and metastasis [2]. In addition, most newly diagnosed GC patients have metastatic diseases, which are still major therapeutic challenges for oncologists [3]. Our previous study also showed that EGFL7 was expressed in gastric carcinoma [4], and it’s high expression was significantly correlated with pathologic characteristics, the clinical progression, the poor prognosis, as well as in other cancers [5,6]. Therefore, EGFL7 was supposed to be a predictive factor for cancer progress and metastasis. But how it functions is still unknown to us, especially in gastric cancer. We employed shRNA to knockdown the expression of EGFL7 in GC cells (BGC823 cells). Our study led to the identification of EGFL7 has a pivotal role in GC metastasis

For the purpose, we used the xenograft cancer model of GC, which could confirm the in vitro study of the effect of EGFL7 in gastric cancer.

Objectives

The objective of this study was to explore the effect of EGFL7 on GC tumorigenesis and metastasis.

Ethical statement

The use of nude mice in this study complies with the Guide for the Care and Use of Laboratory Animals (National Institutes of Health [NIH] publication no. 86–23, revised 1985) and the current Chinese law on the protection of animals (LLSC [LA] 2013- 0010).

Study design

. The nude mice were divided randomly into four groups of five each. There were BGC823, BGC-NC, BGC2-13 and negative control group. BGC823, BGC-NC, and BGC2-13cells were detached and resuspended in PBS. BGC823, BGC-NC, BGC2-13 cells and PBS were subcutaneously implanted into the left upper extremity of nude mice. The mice were executed by method of cervical dislocation after 4-5 weeks, and tumors were removed and weighed. All organs were examined for metastasis. The removed tumors were fixed in 10% formalin, embedded in paraffin, and then cut into 4 μm-thick sections. MVD expression was determined by immunohistochemistry.

Experimental procedures

The nude mice were used as tumor implantation models and each group housed in individually ventilated cages. Then, 0.2 ml of each suspension containing 1×10^8^ cells was subcutaneously injected into the the left upper extremity of each mice, using PBS as the control. Tumor growth was evaluated by measuring tumor diameters with a Vernier caliper every 3 d. Tumor volume (TV) was calculated according to the formula: TV (cm^3^) = d^2^ ×D/2, where d and D represent the shortest and the longest diameters, respectively.

Experimental animals

Twenty female Balb/c nude mice aged 4 to 6 weeks were purchased from the Animal Experimental Centre of the Third Xiangya Hospital, Central South University.

Housing and husbandry

Mice were housed in a temperature (20±1ºC) and humidity (50±5%) controlled room. Each group nude mice were housed in microisolator cages with autoclaved bedding in a specific pathogen-free facility (SPF) with an inverse 12 hours day-night cycle with lights on at 8:00pm. Animals were observed for signs of tumor growth, activity, feeding and pain in accordance with the guidelines of the Research Ethics Committee on Animals, Central South University, China.

All mice were allowed free access to water and a maintenance diet containing in a 12-hour light/dark cycle, with room temperature at 20±1 °C. All cages contained wood shavings, bedding and a cardboard tube for environmental enrichment.

This housing facility is a barrier housing facility, and it has in keeping with national standard《Laboratory Animal—Requirements of Environment and Housing Facilities》(GB 14925—2001). The care of laboratory animal and the animal experimental operation have conforming to《Hunan Administration Rule of Laboratory Animal》,et al)

Sample size

The nude mice were divided randomly into four groups of five each. Mice of negative group was subcutaneously injected into the left upper extremity of PBS serving as control. Mice of BGC823, BGC-NC, and BGC2-13 groups were subcutaneously injected into the left upper extremity with BGC823, BGC-NC, and BGC2-13 suspension cells, respectively.

Allocating animals to experimental groups

N/A

Experimental outcomes

Three primary outcome were analyzed: The tumor weight and volume, livers metastasis of GC cells. One secondary outcome were evaluated: the immune-histochemistry detected of MVD.

Statistical methods

The results were expressed as means ± SD. The tumor weight and volume, and the immunohistochemistry results of MVD of intergroup comparisons were assessed by ANOVA.

For each test, the experimental unit was an individual animal.

Test for normality was performed by Kolmogorov–Smirnov test.

**RESULTS**

Baseline data

The animals’ health status was monitored throughout the experiments by a health surveillance programme according to Federation of European Laboratory Animal Science Associations (FELASA) guidelines. The mice were free of all viral, bacterial, and parasitic pathogens listed in the FELASA recommendations

Numbers analysed

Four tumor-positive samples were from the group of BGC823, with a positive rate of 80% (4/5), and five positive samples were detected in the BGCNC group, with a positive rate of 100% (5/5), and three positive were found in BGC2-13 group, with a positive rate of 60% (3/5)

Twenty nude mice were utilized for this study and 16 were included and completed. Four animals were excluded, including 4 nude mice which were excluded because of tumor-negative.

Outcomes and estimation

Tumor growth was evaluated by measuring tumor diameters with a Vernier caliper every 3 d (Figure A). The mice were executed by method of cervical dislocation after 4-5 weeks. The tumors volume (cm^3^) in the BGC2-13 group were smaller than those in the BGC823 and BGC-NC groups (0.457±0.096 versus 1.428±0.78 and 1.29±0.29, p<0.05) (Figure B). And also the tumors weight (g) in the BGC2-13 group were smaller than those in the BGC823 and BGC-NC groups (0.87±0.13 versus 1.78±0.34 and 1.80±0.19, p<0.05) (Figure C). In addition, ischemic necrosis was observed on the surface of tumors in the BGC2-13 group. MVD was calculated in tumors of the three groups, and the results showed that the average MVD of the tumors in the BGC2-13 group was lower than that in the BGC823 and BGC-NC groups(4±1.2 versus 15±2 and 13±1, p<0.05) (Figure D). Mice livers were observed to determine the metastasis of GC cells. We observed more metastatic cancer cells in live tissues of BGC823 and BGC-NC groups than in live tissues of BGC2-13 group (Figure E).
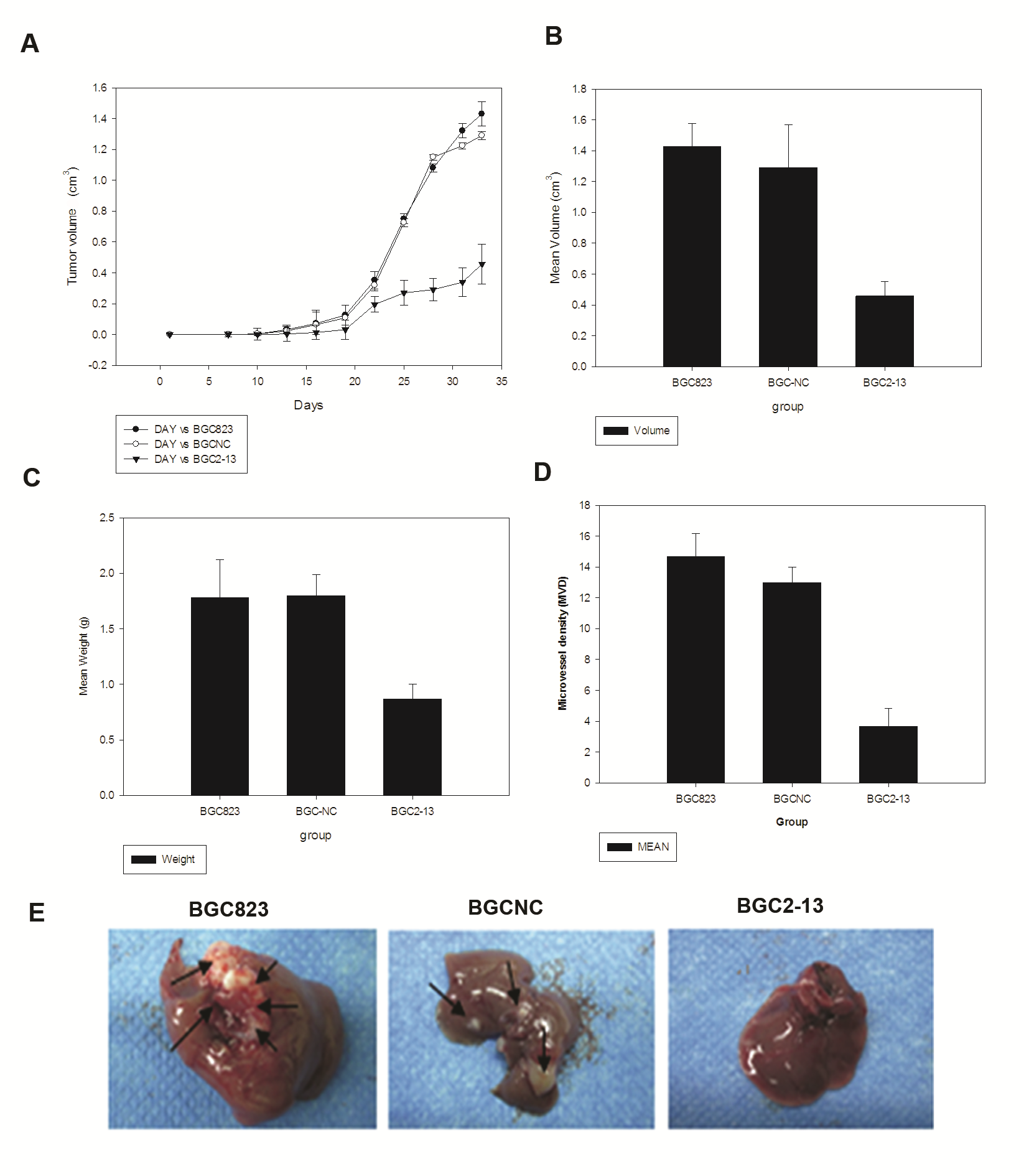


Adverse events

N/A

**DISCUSSION**

Invasion and metastasis are the major obstacles in the treatment of malignancy, and are the major causes of death. Lots of researches were carried out to explore this phenomenon. However, the mechanism in detail under it remains unclear. So investigations about this course are of great importance. In our previous study, high expression of EGFL7 was found to correlated positively with the invasion and metastasis of gastric carcinoma [4]. The alteration of biological behaviors were further confirmed in nude mice. Suppression of the EGFL7 group more significantly decreased in the metastatic cancer cells of the live tissues compared with those in the control groups, indicating that EGFL7 has a critical role in regulating GC metastasis. In addition, we found that reduction of EGFL7 expression decreased the growth of the xenotransplants of these cells in nude mice models, which are similar to the discovery in hepatic carcinoma [5]. As the in vivo study showed that the surface of the tumors exhibited ischemic necrosis with the depletion of EGFL7, indicating that EGFL7 possibly suppresses angiogenesis and slows down the growth of xenograft tumors. Moreover, the average MVD in xenograft tumors of the BGC2-13 group was lower than those in the BGC823 and BGC-NC groups, which consistent with the idea that knocking down EGFL7 could inhibit angiogenesis of tumors, in vivo [7,8].

A limitation of this study is the fact that the numbers of mice in each group, since some mice were failure to form tumors.

Generalisability translation

Establishing the xenograft cancer model of gastric cancer is likely to help us understand the gastric tumorigenesis and metastasis in vivo.

Funding

This work was supported by National Natural Science Foundation of China (No. 81001080) and the Open-End Fund for the Valuable and Precision Instruments of Central South University (No. JJ3121)

**References:**

1. Jemal A, Bray F, Center MM, Ferlay J, Ward E, et al. (2011) Global cancer statistics. CA Cancer J Clin 61: 69-90.

2. Grille SJ, Bellacosa A, Upson J, Klein-Szanto AJ, van Roy F, et al. (2003) The protein kinase Akt induces epithelial mesenchymal transition and promotes enhanced motility and invasiveness of squamous cell carcinoma lines. Cancer Res 63: 2172-2178.

3. Kanat O, O'Neil BH (2013) Metastatic gastric cancer treatment: a little slow but worthy progress. Med Oncol 30: 464.

4. WANG K S, N S, LI J H (2011) Expression of EGFL7 in Gastric Carcinoma and Its Correlation with Tumor Clinical and Pathological Features. WORLD SCI-TECH R&D 33: 650-653.

5. Wu F, Yang LY, Li YF, Ou DP, Chen DP, et al. (2009) Novel role for epidermal growth factor-like domain 7 in metastasis of human hepatocellular carcinoma. Hepatology 50: 1839-1850.

6. Soncin F, Mattot V, Lionneton F, Spruyt N, Lepretre F, et al. (2003) VE-statin, an endothelial repressor of smooth muscle cell migration. EMBO J 22: 5700-5711.

7. CRPC A (2013) EGFL7-Targeting Antibodies Suppress Tumor Angiogenesis. Cancer Discov 3: 1092.

8. Nichol D, Stuhlmann H (2012) EGFL7: a unique angiogenic signaling factor in vascular development and disease. Blood 119: 1345-1352.
